# Mortality Statistics

**Published:** 1874-08

**Authors:** 


### *Chicago Mortality Report for June, 1874.*

MORTALITY IN MONTH OF JUNE, 1874.

| | | | |
|---|---|---|---|
| Accident, by burns | 1 | Croup, membranous | 2 |
| " by being crushed | 1 | Cyanosis | 3 |
| " by drowning | 17 | Debility, general | 8 |
| " by fall | 1 | Delirium tremens | 1 |
| " run over by wagon | 2 | Diarrhœa | 7 |
| " thrown from buggy | 1 | Diphtheria | 2 |
| " thrown from horse | 1 | Dropsy, general | 2 |
| " by scalding | 2 | " ovarian | 1 |
| " by suffocation | 1 | Dysentery | 3 |
| " by railroad | 7 | " chronic | 1 |
| Abscess | 1 | Entero colitis | 4 |
| " lumbar | 1 | Enteritis | 17 |
| Anæmia | 1 | Exhaustion | 1 |
| Apoplexia | 9 | Erysipelas | 4 |
| Bowels, ulceration of | 1 | Fever, congestive | 1 |
| Brain, congestion of | 7 | " intermittent | 1 |
| " inflammation of | 10 | " puerperal | 5 |
| Bronchitis | 6 | " remittent | 2 |
| " capillary | 1 | " scarlet | 14 |
| Cancer of breast | 2 | " typhoid | 7 |
| " lung | 1 | Gangrene | 1 |
| " stomach | 3 | Gastritis | 3 |
| Child birth | 2 | Gastro enteritis | 1 |
| Cholera infantum | 68 | Hemorrhage, internal | 1 |
| " morbus | 2 | Heart, disease of | 6 |
| Consumption | 41 | " organic disease of | 1 |
| Convulsions | 65 | " valvular disease of | 5 |
| " puerperal | 2 | " rheumatism of | 1 |
| Croup | 2 | Hip disease | 1 |

| | | | |
|---|---|---|---|
| Hepatitis | 1 | Peritonitis | 1 |
| Hernia, strangulated | 1 | " puerperal | 1 |
| Hydrocephalus | 9 | Pneumonia | 14 |
| Inanition | 16 | Pleurisy | 1 |
| Illeo colitis | 1 | Pyæmia | 1 |
| Kidneys, Bright's disease of | 7 | Pyo nephritis | 1 |
| " fatty degeneration of | 1 | Rachitis | 1 |
| Laryngitis | 2 | Scrofula | 3 |
| Liver, disease of | 2 | Septicæmia | 1 |
| " cirrhosis of | 1 | Small-Pox | 7 |
| " fatty degeneration of | 1 | Spina bifida | 2 |
| Lungs, congestion of | 4 | Sun stroke | 4 |
| " hemorrhage of | 1 | Suicide | 8 |
| " paralysis of | 1 | Tabes mesenterica | 14 |
| Mania, puerperal | 1 | Teething | 2 |
| Measles | 4 | " and complications | 7 |
| Meningitis | 11 | Tetanus | 1 |
| " cerebro-spinal | 6 | Tumor | 1 |
| " tubercular | 2 | Tonsilitis | 1 |
| Metritis | 1 | Uremia | 1 |
| " puerperal | 1 | Uterus, hemorrhage of | 1 |
| Œdema pulmonum | 2 | Vertebra, caries of | 2 |
| Old age | 9 | Whooping cough | 13 |
| Paralysis | 3 | Total | 542 |

COMPARISON.

| | | |
|---|---|---|
| Deaths in month of June, 1874 | | 542 |
| " " May, 1874 | | 509 |
| Increase | 33 | |
| Deaths in month of June, 1873 | | 658 |
| Decrease | 116 | |

AGES.

| | | | |
|---|---|---|---|
| Under one year | 231 | Forty years to fifty | 39 |
| One year to two | 59 | Fifty " " sixty | 21 |
| Two years to three | 17 | Sixty " " seventy | 15 |
| Three " " four | 6 | Seventy " " eighty | 20 |
| Four " " five | 8 | Eighty " " ninety | 4 |
| Five " " ten | 15 | Ninety " " one hundred | -- |
| Ten " " twenty | 22 | | |
| Twenty " " thirty | 41 | Total | 542 |
| Thirty " " forty | 44 | | |
| Colored | 6 | Males | 296 |
| White | 536 | Females | 246 |
| Total | 542 | Total | 542 |

Married, 131; Single, 411. Total 542

NATIONALITIES.

| | | | |
|---|---|---|---|
| Belgium | 2 | India | 1 |
| Bohemia | 6 | Italy | 1 |
| Canada | 1 | Ireland | 40 |
| Native—Chicago | 73 | Norway | 5 |
| Foreign, " | 231 | Poland | 2 |
| United States, other parts | 72 | Scotland | 6 |
| Denmark | 1 | Sweden | 8 |
| England | 13 | Switzerland | 1 |
| Germany | 64 | Wales | 1 |
| Holland | 3 | Unknown | 11 |

Deaths daily, 18. Mean temperature, 70°. Rain fall, 3.25 inches. 542

MORTALITY BY WARDS, ETC.

| Wards. | No. Deaths. | Wards. | No. Deaths. | Wards. | No. Deaths. |
|---|---|---|---|---|---|
| 1 | -- | 8 | 47 | 15 | 75 |
| 2 | 3 | 9 | 42 | 16 | 37 |
| 3 | 16 | 10 | 9 | 17 | 26 |
| 4 | 8 | 11 | 11 | 18 | 16 |
| 5 | 13 | 12 | 12 | 19 | 1 |
| 6 | 41 | 13 | 8 | 20 | 3 |
| 7 | 61 | 14 | 11 | | 440 |

| | |
|---|---|
| No. of deaths in Wards | 440 |
| Accidents | 34 |
| Bridewell | 1 |
| County Hospital | 12 |
| Foundlings' Home | 25 |
| Home for Friendless | 2 |
| Hospital Alexian Brothers | 1 |
| Hospital for Women and Children | 2 |
| Mercy Hospital | 6 |
| Hahneman Hospital | 1 |
| Protestant Orphan Asylum | 2 |
| R. R. Depot | 1 |
| St. Joseph's Orphan Asylum | 1 |
| St. Joseph's Hospital | 3 |
| Small Pox " | 1 |
| St. Luke's " | 2 |
| Suicide | 8 |
| Total | 542 |

CASES OF SMALL-POX REPORTED DURING JUNE, 1874.

| Wards. | No. Cases. | Wards. | No. Cases. | Wards. | No. Cases. |
|---|---|---|---|---|---|
| 1 | -- | 8 | -- | 15 | 8 |
| 2 | -- | 9 | -- | 16 | 6 |
| 3 | -- | 10 | -- | 17 | 3 |
| 4 | 2 | 11 | -- | 18 | 2 |
| 5 | -- | 12 | -- | 19 | 1 |
| 6 | 9 | 13 | 1 | 20 | -- |
| 7 | 3 | 14 | -- | Recent arrival | 1 |
| Total | | | | | 36 |

| | |
|---|---|
| Cases reported during June, 1874 | 36 |
| " " " May, 1874 | 56 |
| Decrease | 20 |
| Cases reported during June, 1873 | 149 |
| Decrease | 113 |

*To Editor Journal :*

In this report you will see that compared with last month the increase was 33 deaths. With the increase of temperature the rate of death always increases; the increase being among children under five years of age. The temperature for this month was 22° higher than for the last month, and 26° higher than for the corresponding month of last year. At that time an epidemic of cholera was raging, while this year no epidemic of any kind is present. With the increase of temperature the rate of mortality, especially among children, may be expected to increase four-fold compared with the present rate. I cannot too strongly urge the profession to use every endeavor to have the families in their charge take unusual precautions in the care of their children, for from present indications the death rate will be unusually high this summer, and too much care cannot be taken in the management of children.

BEN. C. MILLER,
*Sanitary Supt.*

